# Environmental Exposures and Hearing Loss

**DOI:** 10.3390/ijerph17134879

**Published:** 2020-07-07

**Authors:** Rita Rosati, Samson Jamesdaniel

**Affiliations:** 1 Institute of Environmental Health Sciences, Wayne State University, Detroit, MI 48202, USA; ad6628@wayne.edu; 2 Department of Family Medicine and Public Health Sciences, Wayne State University, Detroit, MI 48201, USA

**Keywords:** hearing loss, ototoxicity, environmental exposures, noise, lead, BTEX, organic solvents

## Abstract

Pollutants that contaminate the natural or built environment adversely affect the health of living organisms. Although exposure to many of them could be avoided or minimized by careful preventive measures, it is impossible to totally avoid exposure to all pollutants. Ototraumatic agents, such as noise, chemicals, and heavy metals, are pervasive pollutants, mostly produced by human activity, and are critical factors in inducing acquired hearing loss. More importantly, exposure to these pollutants often occurs concurrently and, therefore, the synergistic interactions potentiate auditory dysfunction in susceptible individuals. Epidemiological studies have provided compelling data on the incidence of auditory dysfunction after exposure to a number of ototraumatic agents in the environment, while animal studies have offered crucial insights for understanding the underlying molecular mechanisms. Together, they provide a framework for developing effective interventional approaches for mitigating the adverse impacts of environmental or occupational exposure to ototraumatic agents. This article provides a brief overview of the common pollutants that cause hearing loss.

## 1. Introduction

The major risk factors of acquired hearing loss, the third most common chronic physical condition among American adults, after hypertension and arthritis, include environmental ototraumatic agents, such as noise, chemicals, and heavy metals. Though the acquired hearing loss induced by these agents is usually insidious and not catastrophic, the consequences are serious because they: (1) affect the quality of life and productivity; (2) are linked to cognitive decline, dementia, Alzheimer’s Disease, and depression; (3) have devastating effects in children by affecting speech and language development, education, and social integration; (4) impose a huge economic burden (e.g., 34% of veteran disability benefit claims are for hearing loss and tinnitus). As the prevalence of hearing loss worldwide is estimated to double by 2050 to include over 900 million people (World Health Organization, 2018), there is a critical need to identify emerging risk factors that pollute the environment and discover effective prevention and treatment strategies.

## 2. Noise

Noise, which includes any unwanted or disturbing sound, is a pervasive pollutant in modern society. Exposure to loud noise from recreational, environmental or occupational sources leads to noise-induced hearing loss, a common type of acquired hearing loss [1]. Noise exposure at moderate levels can induce a temporary shift in hearing thresholds, caused by reversible damage to the stereocilia of hair cells, while exposure to loud noise leads to permanent shift in hearing thresholds, due to irreversible damage to cochlear hair cells [2]. The potential for hearing damage depends on the type of noise and the duration of exposure because noise-induced hearing loss is usually related to the energy of the exposed noise. The characteristics of hearing loss in people exposed to continuous noise (an uninterrupted sound level that varies less than 5 dB during the period of observation), may vary from those exposed to intermittent noise (a continuous noise that persists for more than 1 s and interrupted for more than 1 s), or impulse noise (a change of sound pressure of 40 dB or more within 0.5 s with a duration of less than 1 s). The effects of noise on the cochlea are diverse as they may result in outer hair cell loss, reduced blood flow in the basal region, rupture of tight cell junctions, excitotoxicity of VIII nerve fibers, cochlear synaptopathy, or loss of inner hair cells and VIII nerve fibers [3,4,5,6,7]. In addition to hearing loss, exposure to loud noise can lead to hearing disorders, such as tinnitus, recruitment, and hyperacusis.

## 3. Heavy Metals

Heavy metals are naturally found in the earth’s crust [8]. They are released in the environment by activities, such as mining, metal working, and coal burning. Upon absorption they accumulate in soft tissues and bones and alter cellular homeostasis [8,9]. Long term exposure to heavy metals, such as lead, cadmium, cobalt, arsenic, and mercury, can cause auditory dysfunction [10,11,12,13,14]. Particularly, exposure to cadmium and lead damages the sensory receptor cells in the cochlea [15,16] and induces oxidative stress in several other organs [17,18,19]. As simultaneous exposures to multiple ototoxicants have a synergistic effect in the inner ear, the co-exposure of heavy metals with other ototraumatic agents can exacerbate the hearing loss [20,21,22,23]. Sources of lead include lead-based paints in older homes, batteries, solder, pipes, pottery, roofing materials, and some cosmetics. Sources of cadmium include burning fossil fuels (such as coal or oil), the incineration of municipal waste, cigarette smoke, contaminated food (shellfish, vegetables), and ambient air in industrialized urban areas [24]. As with noise-induced and ototoxic hearing loss, because the sensory receptor cells in the inner ear cannot regenerate, the hearing loss induced by these ototoxicants is permanent.

## 4. Ototoxic Chemicals

Among the multitude of chemical substances in the environment, a group of compounds that are known to induce ototoxicity are organic solvents [25,26,27]. Occupational or environmental exposure to organic solvents, such as toluene, styrene, xylene, and ethyl benzene, can significantly impair auditory perception [28,29]. Many of these organic solvents target the mid-frequency region of the cochlea [30,31]. Exposure to styrene induces oxidative stress in the inner ear. The generation of superoxide radical in the organ of Corti, spiral ganglion neurons, and stria vascularis and increased levels of 8-Isoprostane, a biomarker of lipid peroxidation, in the stria vascularis and spiral ganglion neurons have been reported after styrene exposure [32]. Exposure to BTEX (Benzene, Toluene, Ethylbenzene, and Xylene), a cocktail of highly soluble and volatile organic compounds naturally occurring in crude oil and petroleum products, is also a major risk factor for hearing loss [33,34], as ototoxic levels of BTEX can be present in both indoor and outdoor environments [35]. Emissions from motor vehicles, petrol stations, and refineries are major sources of BTEX in the outdoor environment. BTEX is estimated to constitute up to 60% of non-methane volatile organic compounds in the urban atmosphere [36]. In the indoor environment, BTEX is found in consumer goods, such as paints and lacquers, thinners, rubber products, adhesives, inks, cosmetics, and pharmaceutical products. The degree of hearing loss associated with exposure to these compounds varies with organic solvent type and exposure level.

## 5. Conclusions

Most of the studies that investigate ototraumatic agents in the environment assess their ototoxic properties by evaluating them individually. The results obtained from such isolated-exposure studies are mainly those considered for determining exposure limits and designing hearing conservation programs. Very little data exist on the potential synergistic effects of combined exposure to environmental ototraumatic agents, which is the most common type of exposure occurring in the real-world. There is, therefore, an urgent need to reassess the regulatory standards that govern exposure levels of ototraumatic agents by taking in to consideration the ototoxic potential of combined exposures and by delineating the underlying molecular mechanisms. In the absence of such knowledge, effective interventional strategies to prevent and treat ototoxicity induced by environmental risk factors will remain elusive.
